# Human iPS cell derived RPE strips for secure delivery of graft cells at a target place with minimal surgical invasion

**DOI:** 10.1038/s41598-021-00703-x

**Published:** 2021-11-02

**Authors:** Mitsuhiro Nishida, Yuji Tanaka, Yo Tanaka, Satoshi Amaya, Nobuyuki Tanaka, Hirofumi Uyama, Tomohiro Masuda, Akishi Onishi, Junki Sho, Satoshi Yokota, Masayo Takahashi, Michiko Mandai

**Affiliations:** 1 Laboratory for Retinal Regeneration, RIKEN Center for Biosystems Dynamics Research, 2-2-3 Minatojima-minamimachi, Chuo-ku, Kobe, 650-0047 Japan; 2 Division of Medicine, Interdisciplinary Graduate School of Medicine and Engineering, University of Yamanashi, Kofu, Japan; 3 Laboratory for Integrated Biodevice, RIKEN Center for Biosystems Dynamics Research, Kobe, Japan; 4 Department of Ophthalmology, Kobe City Eye Hospital, Kobe, Hyogo Japan; 5 Vision Care Inc., Kobe, Hyogo Japan; 6 Vision Care Cell Therapy Inc., Kobe, Hyogo Japan

**Keywords:** Cell delivery, Regenerative medicine, Tissue engineering

## Abstract

Several clinical studies have been conducted into the practicality and safety of regenerative therapy using hESC/iPSC-retinal pigment epithelium (RPE) as a treatment for the diseases including age-related macular degeneration. These studies used either suspensions of RPE cells or an RPE cell sheet. The cells can be injected using a minimally invasive procedure but the delivery of an intended number of cells at an exact target location is difficult; cell sheets take a longer time to prepare, and the surgical procedure is invasive but can be placed at the target area. In the research reported here, we combined the advantages of the two approaches by producing a quickly formed hiPSC-RPE strip in as short as 2 days. The strip readily expanded into a monolayer sheet on the plate, and after transplantation in nude rats, it showed a potency to partly expand with the correct apical/basal polarity in vivo, although limited in expansion area in the presence of healthy host RPE. The strip could be injected into a target area in animal eyes using a 24G canula tip.

## Introduction

Over the past decade, for retinal degeneration, one therapeutic approach which has attracted considerable interest is the use of ES/iPSC-derived retinal pigment epithelial cells (RPE) for cell-based regenerative therapy^1–8^. Clinical studies have indicated that this therapeutic approach is generally safe and has some possible efficacy. There have been two major approaches taken in these clinical studies: one using cell suspensions, and the other using an RPE sheet. Each approach has advantages and disadvantages. The transplantation of cell suspensions can utilize ready-to-use cell stocks out of a tube or from a short-term recovery culture, and such cells can be transplanted with minor surgical intervention, although controlling the cell delivery to the exact target area can be difficult, and transplanted cells often leak into the vitreous to form an epiretinal membrane (ERM)^5,8^. The use of an RPE sheet allows visual confirmation of placement, but the preparation of a sheet requires considerable time and cost, and involves an invasive surgical procedure requiring a large incision in the sclera and neural retina.

We conducted a clinical trial using transplantation of an autologous iPSC-RPE sheet. The transplanted sheet looked rolled immediately after the transplantation, but eventually expanded to cover approximately 1.5 times the area of the original RPE sheet, possibly by flattening of the sheet with possible migration and proliferation of the cells ^4,9^. This observation gave us an idea that even a strip form of RPEs, that could easily be injected subretinally, may expand to cover a larger area, similar to a small sheet transplantation. This approach may enable precise graft placement at the intended area with minimal back-flow leakage into the vitreous and may allow for supplementation of RPEs in any desired multiple areas of the ocular fundus.

A recent study investigated the mechanics of microtissue morphology by examining the relationship between surface coverage, tissue contractility, and adhesion strength ^10^. These researchers found that cells with balanced adhesiveness and contractility spontaneously generated spheroid-like tissue structures on a concave surface with a small curvature. Based on this finding, we employed a groove surface with a small curvature of the bottom edge to generate a strip of tissue. Although the fabrication of small tissues requires special techniques such as photolithography, we introduced a fabrication technique combining polydimethylsiloxane (PDMS) soft lithography^11,12^ and simple three-dimensional (3D) printing ^13^.

In the present study, we prepared a PDMS-based culture device with narrow grooves, to investigate the formation of hiPSC-RPE strips. We then confirmed that hiPSC-RPE cells could expand from a strip that was plated on a dish, to produce RPEs with characteristics like those before a strip formation. We also investigated whether a strip of hiPSC-RPE could be successfully injected into animal eyes and has a potency to expand with the correct polarity.

## Results

### Preparation of strip-form hiPSC-RPEs

**Figure 1:**
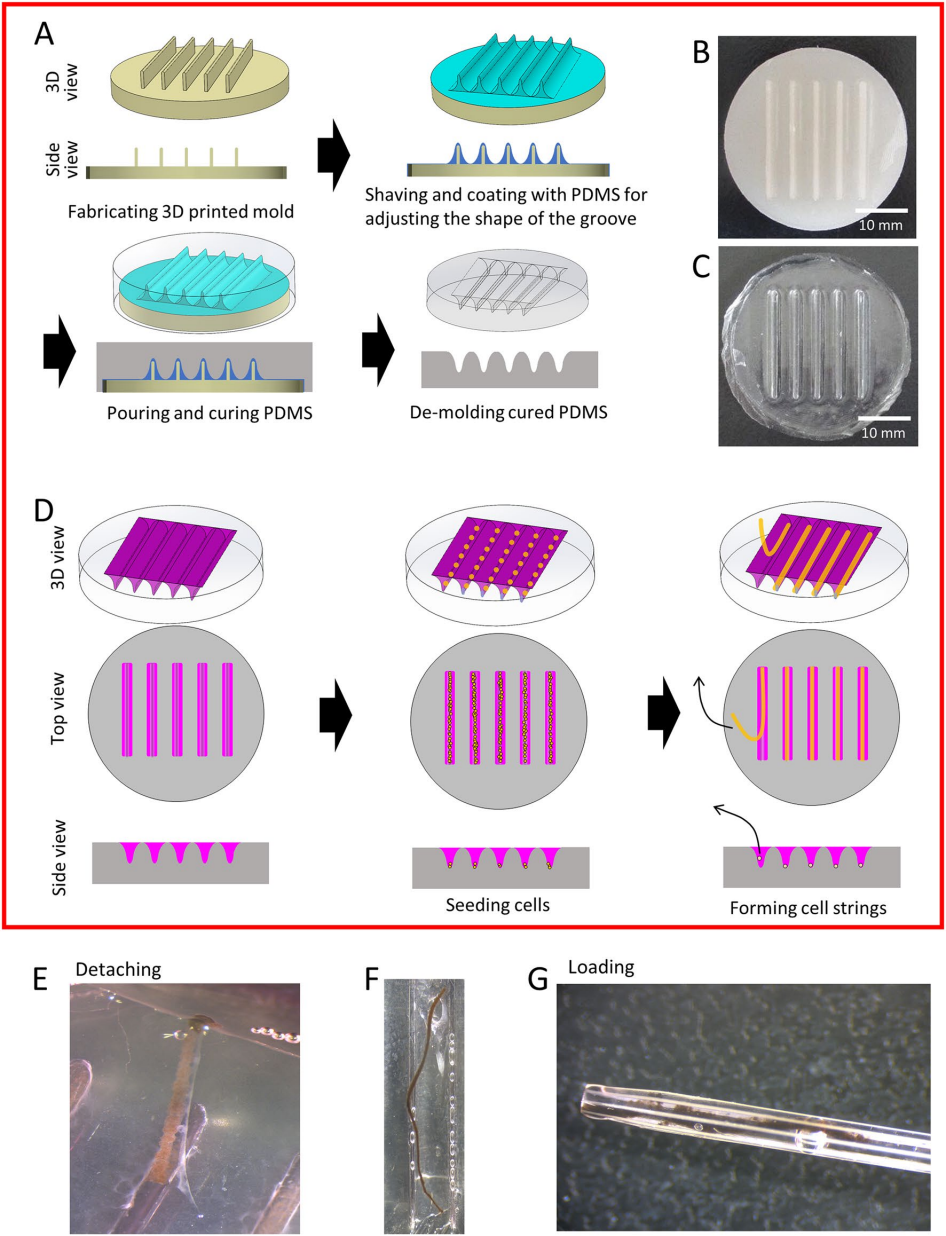
Manufacturing of a strip-form hiPSC-RPE. **(A)** Fabrication process of polydimethylsiloxane (PDMS)-based culture device for the formation of RPE-strips. **(B)** 3D printed mold. **(C)** PDMS-based culture device with grooves of 19.5 mm length, 1 mm width, and 1.6 mm depth. The PDMS mixture was poured over the mold, degassed, and then placed in an 80 °C oven for 3 h. See also Supplemental Fig. 1 for detailed data. **(D)** Procedure for making cell strips. RPE cells were seeded in each groove, which were left to form strips for two days. **(E–G**) Representative images of hiPSC-RPE cells in a strip form that can be easily released from the mold **(E,F)** and loaded in a 24G cannula **(G)**.

The protocol for strip preparation using a PDMS-based culture device is summarized in Fig. 1 (see also Figure S1). Human iPSC-RPE cells from the cell line 253G1 were suspended and seeded in each groove in “RPE Maintenance medium”, “RPE cell sheet medium” (F10 medium containing 10% fetal bovine serum) or “mixed medium” with 10 µM Y-27632, an inhibitor of the protein serine/threonine kinase 160ROCK. The strips appeared to be easily collapsed in the sheet medium, but a strip structure was well retained in the maintenance medium and mixed medium (Figure S2A and B). Different numbers of cells (4.5 × 10^5^, 1.5 × 10^5^, and 5 × 10^4^) were seeded on the culture device, and it was observed that the first two conditions could lead to the formation of strips (Figure S2C). An example of a strip formed with 4.5 × 10^5^ of hiPSC-RPEs is shown in Fig. 1E,F. The strips were detached from the groove and loaded on 24G intravenous cannula (Fig. 1G).

### Optimization of Y-27632 concentration for hiPSC-RPE strips

**Figure 2:**
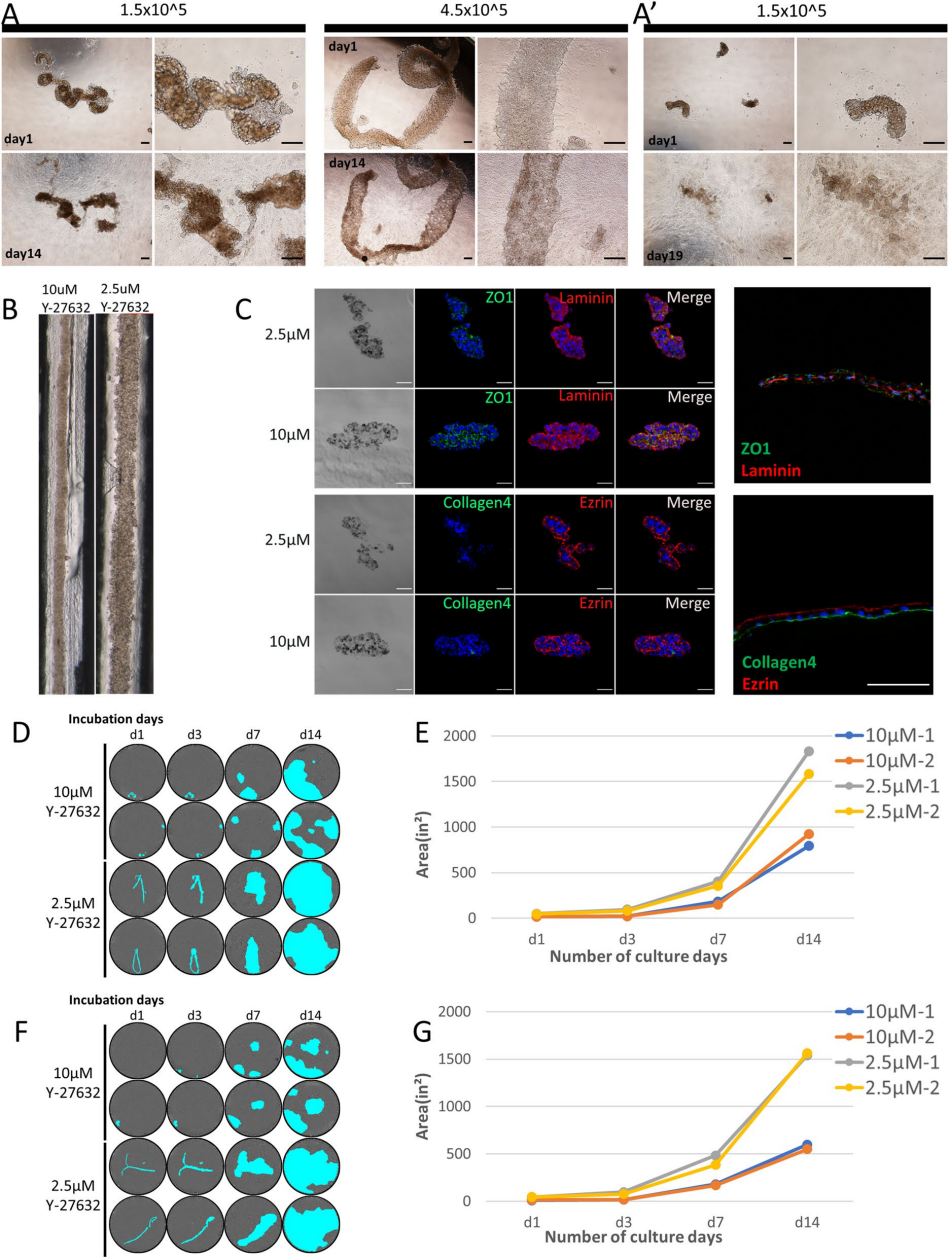
Effect of number of starting cells and Y-27632 concentration on hiPSC-RPE strip formation. **(A)** The appearance of strips on day 4, formed with 1.5 × 10^5^ or 4.5 × 10^5^ RPE cells placed on a dish and photographed on the next day (day 1) and 14 days later (day 14). RPE cells expanded from each strip; the strip formed from 1.5 × 10^5^ cells were losing its initial shape after 14 days, while that formed from 4.5 × 10^5^ cells still retained the initial shape. See also Supplemental Fig. 4. **(A’)** Fragmented strips almost lost their initial shapes after 14 days of cell expansion. **(B)** Appearance of a hiPSC-RPE strip in a groove on day 2 after seeding RPEs (2 × 10^5^) with 10 µM or 2.5 µM Y-27632. **(C)** Sectional view of a hiPSC-RPE strip formed from either 10 µM or 2.5 μM Y-27632. ZO-1 was expressed between the cells, the basal marker laminin was distributed over the cell surfaces, and the apical marker ezrin was localized on the surface of segmented cell clumps. Collagen type IV was absent from the strips. The right panels show the control staining of the same antibody sets on an RPE sheet. **(D–G)** RPE expansion from hiPSC-RPE strips formed from 10 µM or 2.5 μM Y-27632. An RPE strip was placed on a non-coated well and the cell-present area was imaged using IncuCyte and marked in cyan. **(D,F)** Area of cell occupation at each time point. **(E,G)** Day 2 RPE-strip **(D,E)** and day 3 RPE-strip **(F,G)** showed the same tendency. Scale bars 200 µm **(A,A’)**, 50 µm **(C)**.

The strips formed by 4.5 × 10^5^ and 1.5 × 10^5^ cells were plated in wells. Both types of strips showed an expansion of RPE cells on the non-coated plates. The strips formed with 1.5 × 10^5^ cells lost their initial shape faster than the ones formed with 4.5 × 10^5^ cells, which still retained most of their initial form after two weeks (Fig. 2A and Figure S2D). Therefore, we fixed the initial cell number as 1.5–2 × 10^5^ cells. The initial strip forms were mostly lost when the strip was fragmented (Fig. 2A’).

In our preliminary experiments (Figure S3A), the concentration of Y-27632 appeared to affect the strip texture, with a higher concentration making the strip tighter. We tested the effects on strip formation of concentrations of Y-27632 of 0, 1, 2, 2.5, and 10 μM. hiPSC-RPE (M8 Line) cells were able to form strips at a concentration of 2 μM or higher, but at 0 and 1 μM Y-27632, the strips were not well formed. When plated, strips formed with 10 μM Y-27632 detached easily from non-coated wells, took a longer time to attach to 24-well plates, and were delayed in expansion compared to strips formed with 2–2.5 μM Y-27632 (Figure S3B,C).

We compared strips made with either 2.5 μM or 10 μM Y-27632 (Fig. 2B–G). A strip made with 2.5 μM Y-27632 was visually flat and loose, while one formed with 10 μM Y-27632 looked more tensed (Fig. 2B).

The strip sections were immunostained for tight junction marker ZO-1, and polarity markers human Ezrin (apical), Laminin, and human Collagen type IV (basal) (Fig. 2C). In both type of strip, the tight junction marker ZO-1 was expressed between the cells, and the basal marker Laminin was distributed on the cell surfaces, while the apical marker Ezrin appeared to be localized on the surface of segmented cell clumps. Another basal marker, Collagen type IV, was not clearly expressed, indicating that cell polarity was not clearly defined in these RPE strips.

Cell expansion after plating in non-coated wells was compared between the strips formed with 2.5 and 10 μM Y-27632, using strips from day 2 (Fig. 2D,E) and day 3 (Fig. 2F,G). The strips formed with 2.5 μM Y-27632 tended to attach immediately after plating and start expanding, while the strips formed with 10 μM Y-27632 tended to crumple and moved about for a couple of days before they settled and started expansion.

### Reproducibility of strip formation using another hiPSC-RPE line

**Figure 3:**
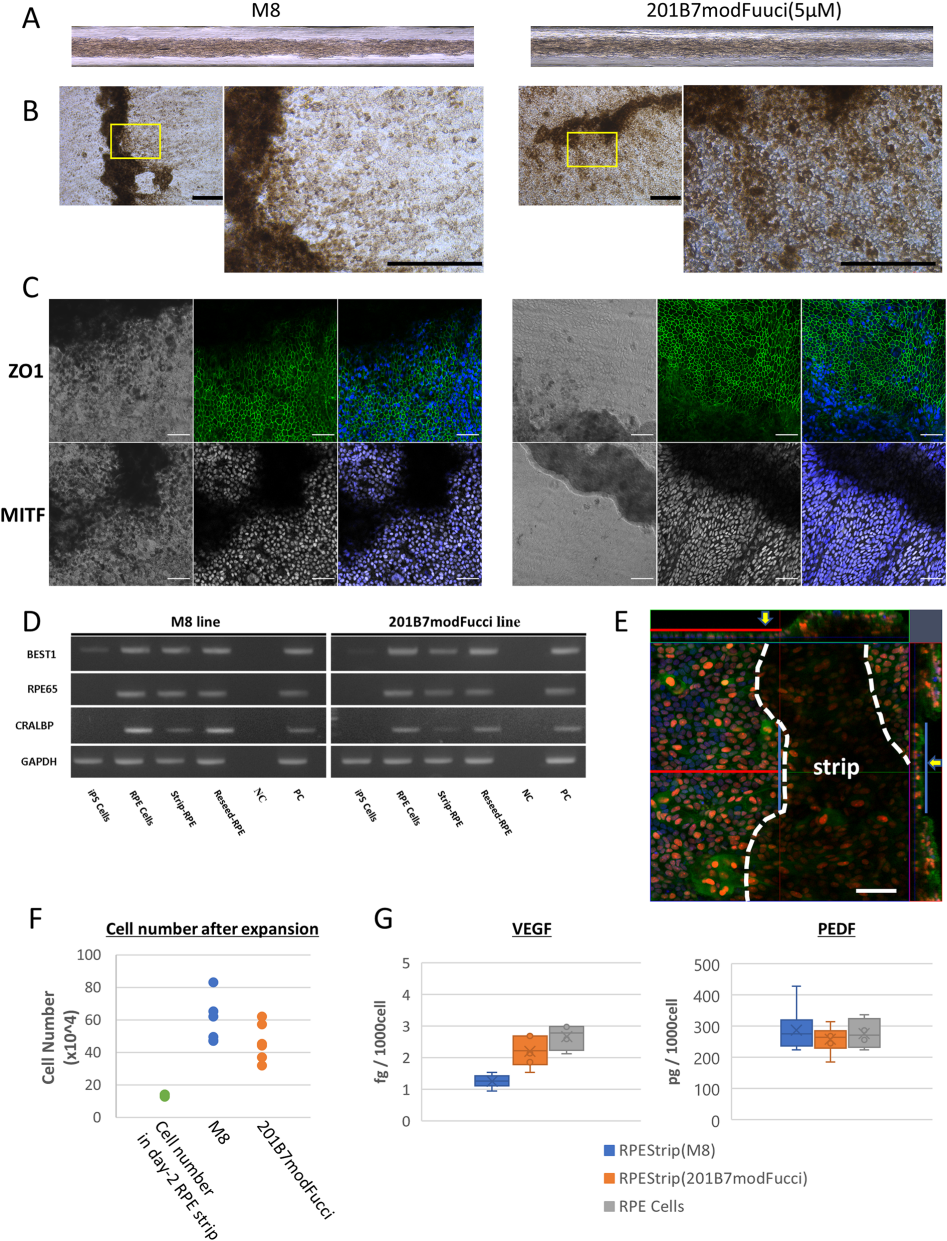
Reproducibility of hiPSC-RPE strip formation using different hiPSC lines (M8 and 201B7modFucci). **(A)** hiPSC-RPE strips were formed using two hiPSC lines, but the 201B7modFucci line required 5 μM or higher concentration of Y-27632 to stably form a strip, which was also optimal for adhesion and expansion (see Supplemental Fig. 3). **(B)** A strip formed with each of M8 (see Supplemental Fig. 4) and 201B7modFucci line (see Supplemental Fig. 5) was adherent to and well expanded on the plate, presenting a characteristic cobblestone-like appearance and pigmentation of RPEs. **(C)** RPE cells expanded from the strip-expressed tight junction marker ZO-1 and the RPE marker MITF. **(D)** RPE marker gene expression in the cells before strip formation; of the strips; and after expansion from the plated strips of each hiPSC line. **(E)** RPE cells were observed to expand from a strip in a single layer. **(F)** Total cell number on a plate after placing a strip and expansion of each hiPSC-RPEs (n = 6 strips for each line). The number of day 2 RPE Strips formed with 2 × 10^5^ RPEs are plotted for comparison (n = 4 strips). **(G)** Expanded RPEs from the strip secreted VEGF and PEDF at a level similar to that of RPEs in culture before strip formation (RPE strip; n = 6 wells, RPE cells; n = 4 wells). Scale bars 200 µm **(B)**, 50 µm **(C,E)**.

We tested whether another hiPSC line could form strips. We induced RPE differentiation using the 201B7modFucci line and compared the differentiated RPEs with hiPSC(M8)-RPEs. The hiPSC(201B7modFucci)-RPEs also formed strips similar to hiPSC(M8)-RPEs, but the 201B7modFucci line required 5 μM or higher concentration of Y-27632 to stably form strips (Fig. 3A). The optimal Y-27632 concentration for adhesion/expansion assay was also 5 μM for hiPSC(201B7modFucci)-RPE strips (Figure S3D and E). The cells expanded from the strips of both hiPSC lines showed characteristic pigmentation and a cobblestone-like appearance of RPE cells and expressed ZO-1 and an RPE marker microphthalmia transcription factor (MITF) (Fig. 3B,C). The expression of the RPE-specific marker genes *BEST1*, *RPE65*, and *RLBP1* was also confirmed by polymerase chain reaction (PCR) in each stage of hiPSC-RPEs before strip formation, in the strip forms, and after expansion from the strips (Fig. 3D). The RPE cells were observed to expand from the strip in a monolayer manner on a plate (Fig. 3E). In order to determine whether the expansion from the strip involved RPE proliferation, the total number of cells 14 days after placing the strip on a plate was compared with the initial cell number in day 2 strips formed from 2 × 10^5^ hiPSC-RPE cells. The total number of expanded cells and the cells in the remaining strip was 5.28 × 10^5^ on average, which was approximately four times the average cell number in the day 2 strip (1.36 × 10^5^), indicating that RPE proliferation contributed to the cell expansion from the strips (day 2 4 strips; expansion from 6 hiPSC(M8)-RPE strips; from 6 hiPSC(201B7modFucci)-RPE strips) (Fig. 3F). The secretion of VEGF and PEDF by the expanded cells and the remaining strip at the same time point were also tested 14 days after placing the strip. Expanded hiPSC-RPEs secreted the same amount of vascular endothelial growth factor (VEGF) and pigment epithelium-derived factor (PEDF) as the cells that were originally used for strip formation (hiPSC(M8)-RPE n = 6 wells and hiPSC(201B7modFucci)-RPE n = 6 wells; pre-strip RPEs of both lines as a control, n = 4 wells) (Fig. 3G).

### Replacement of RPEs by mimicked hiPSC-RPE transplantation by cell suspension or an RPE-strip in the in vitro RPE damaged model

**Figure 4:**
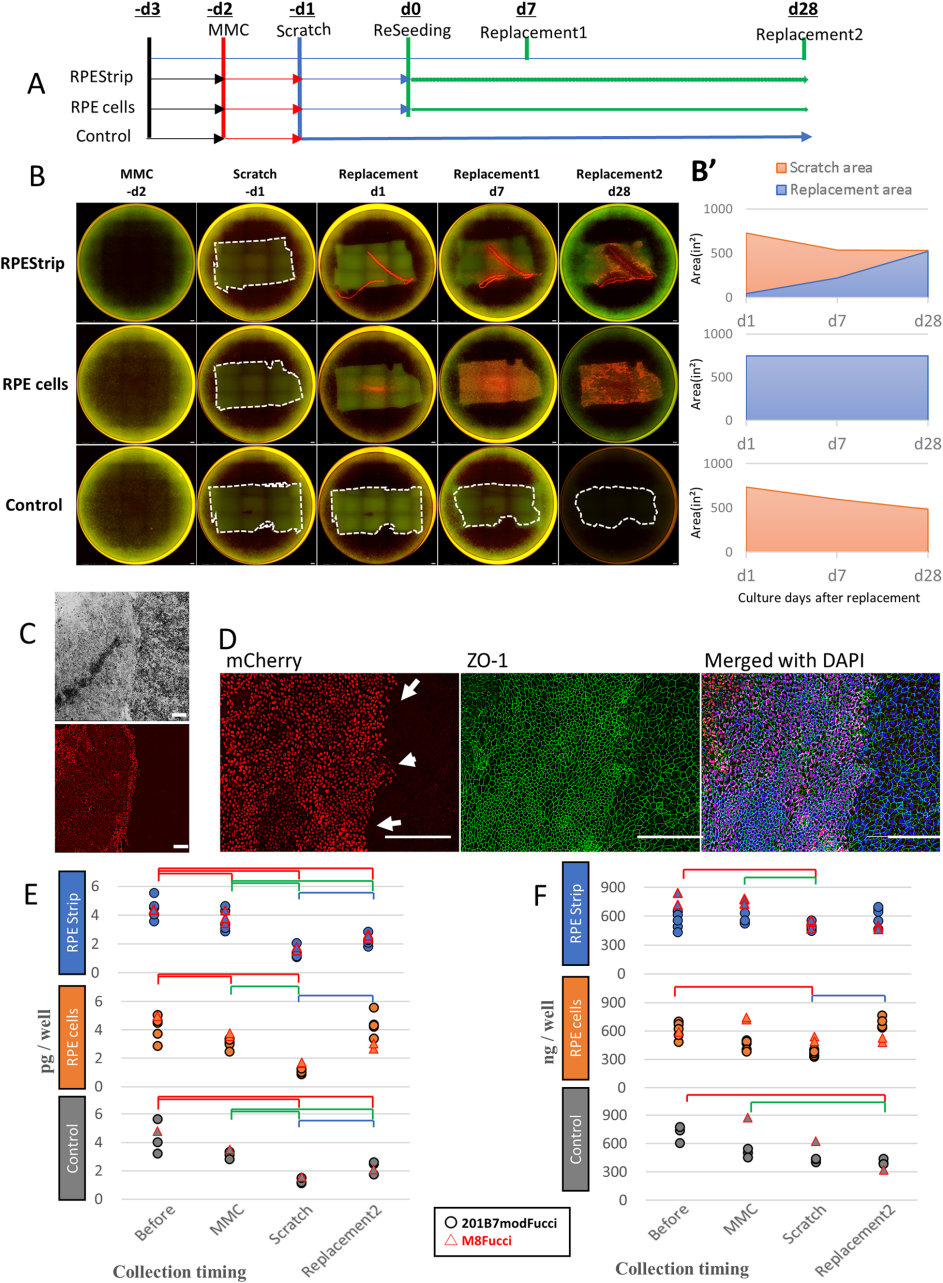
hiPSC-RPE strip replaced the damaged RPE defect area in the in vitro transplantation model. **(A)** An in vitro disease model and transplantation. Pathological RPE degeneration was mimicked in vitro by treating RPE cells with MMC (-d2) and creating a defect area with a scratch (-d1), followed by plating of either single hiPSC-RPE cells (RPE cells) or a hiPSC-RPE strip (M8Fucci2 hiPSC-RPE or 201B7modFucci hiPSC-RPE; see Supplemental Fig. 5) (RPE strip) over the defect area (d0: ReSeeding). Cell occupancy was evaluated at two time points on day 7 (Replacement 1) and on day 28 (Replacement 2). **(B)** Expansion of plated hiRPE cells over the RPE defect area was monitored by mCherry expression in the M8Fucci2 hiPSC-RPEs. The initial scratched area and the defect area in the control well are marked by dotted lines. **(B’)** Panels show the averaged area of RPE defect after scratch (orange) and the refilled area (light blue) by RPE suspension or RPE expansion from the strip (n = 5 wells for cell suspension and strip, n = 3 wells for scratch only control). **(C)** RPE cells expanded from the strip express mCherry at the G0–G1 cell cycle phase and seemed to stop expansion when in contact with pre-existing MMCs, making a border between the two populations. **(D)** A border between the pre-existing MC-treated RPEs and newly plated RPEs was identified by the expression of mCherry and the size of the RPE cells (arrows). MMC treated RPEs were generally larger than RPEs expanded from the RPE-strip, as shown by ZO-1 staining. ZO-1 expression was observed throughout the pre-existing and “transplanted” RPEs, including the cells at the border. E, F: Secretion of VEGF **(E)** and PEDF **(F)** in each well at initiation (-d3), after MMC (-d2), after a scratch (-d1), and after with or without plating hiPSC-RPE cells or strips. Results from 2 separate experiments, one using M8Fucci2 hiPSC-RPE and the other, 201B7modFucci hiPSC-RPE, are plotted. Bars indicate significant changes in secretion (*p* < 0.05, Wilcoxon test) from before MMC (red), after MMC (green), and after a scratch (blue), to the indicated time point (control n = 4, RPE-strip n = 8, RPE cells n = 7, n indicates the total number of samples in 2 independent experiments).

In order to investigate the way in which transplantation of hiPSC-RPE strips or cells could compensate for damaged RPEs, we designed an in vitro model of hiPSC-RPE transplantation in the RPE-damaged environment, as shown in Fig. 4A. In brief, an hiPSC-RPE (M8 line) culture in a non-coated 24 well plate was treated with Mitomycin C (MMC) to suppress proliferation, and the central part was scratched on the next day. On the following day, an RPE strip was placed on the scratched portion, or suspended cells (M8Fucci2 line or 201B7modFucci line) were seeded over the scratch, and filling or replacement by “transplanted” RPEs was monitored using IncuCyte (Fig. 4B).

In the control wells with scratch but without cell supplementation, MMC-treated pre-existing RPEs gradually expanded to slowly decrease the defect area. Transplanted RPE cells in suspension rapidly occupied the area of the defect. Cells in RPE strips expanded from the strip until they contacted pre-existing RPEs, at which point the cell expansion from the strip stopped, apparently due to contact inhibition. The average sizes of the defect area and the area occupied by the transplanted RPEs are shown 1, 7, and 28 days after in vitro transplantation in Fig. 4B’ (strip and suspension; n = 5 wells, control; n = 3 wells). With RPE strips, the border between the pre-existing RPEs and transplanted RPEs was identified by the expression of mCherry by the transplanted M8Fucci2 cells or 201B7modFucci hiPSC- RPEs, and by the size of the RPE cells; MMC treated, non-dividing RPEs were generally larger than those expanded from transplanted RPEs (Fig. 4C). The presence of the tight junction marker ZO-1 was observed throughout both the pre-existing and transplanted RPE populations, without interruption across the border between the two populations (Fig. 4D).

The secretion of VEGF (Fig. 4E) and PEDF (Fig. 4F) was also monitored, to estimate the overall RPE function in each well. VEGF secretion was significantly reduced after MMC and a scratch, and was increased after 28 days, in all groups. Following transplantation of a cell suspension, VEGF secretion was almost comparable to that before MMC treatment. PEDF secretion was reduced after MMC treatment and a scratch but following treatment with a strip transplantation, showed no difference after 28 days from the levels produced before MMC. PEDF secretion was significantly increased after 28 days from those after a scratch with cell transplantation but continued to decrease with no cell replacement (n = total number of wells in 2 independent experiments; control n = 4, RPE-strip n = 8, RPE cells n = 7).

Additionally, we confirmed if proliferation of expanding RPE cells from the strip was contact dependent using a similar setting but without MMC treatment on pre-plated RPE. We fixed the RPE strips at three different time points after placing on a plate with or without pre-plated RPE and immunostained them with the proliferation marker Ki67 (Figure S4). Ki67 positive cells were observed around the strip and the margin of the scratched pre-plated cells on day 7 but became sparse by the time the cells reached confluency (n = 2 for each condition).

### hiPSC-RPE strip transplantation in nude rats

**Figure 5:**
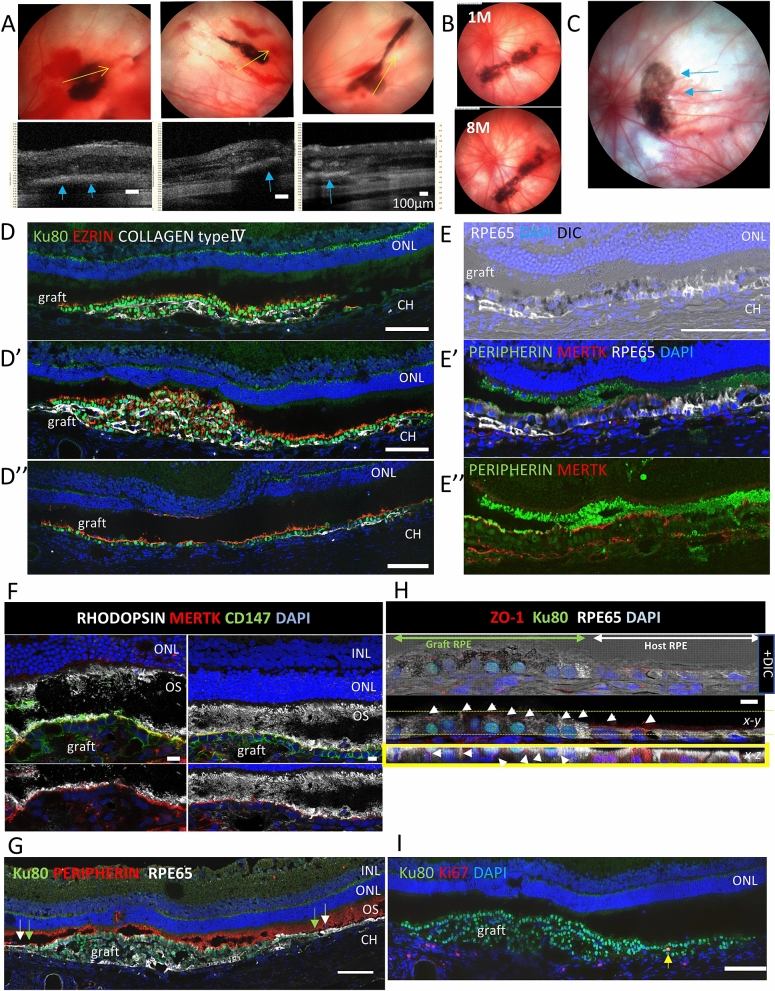
hiPSC-RPE strip transplantation in a nude rat. **(A)** Three representative color fundus images (top) and a sectional view (bottom) taken using optic coherent tomography (OCT) of the graft RPE-trip at the yellow arrow line in the top images 6 weeks after transplantation in albino nude rats. An RPE-like reflex was consistently observed at the flat sheet like area on OCT (blue arrows). **(B)** Color fundus images of a nude rat eye after hiPSC-RPE strip transplantation 1 and 8 months after transplantation. **(C)** A hiPSC-RPE strip graft with sheet like expansion (arrows). **(D,D’’)** Histology of the eye in C. Expression of apical (human Ezrin)/ basal (human Collagen IV) markers on hiPSC-RPE strip graft. The correct polarity was observed in the graft RPE cells in top layer **(D)**, a part of the top layer and the expanding monolayer **(D’)** and the expanded monolayer **(D”)**. **(E,E’’)** Pigmented graft RPE cells express human MERTK on the apical side and contacts Peripherin positive outer segments of host photoreceptors. **(F)** CD147 positive graft human RPE cells express human MERTK on the apical side and contact rhodopsin positive outer segments of host photoreceptor cells. **(G)** The top layer of multilayered graft RPE strip lines up continuously with host RPE cells and contact outer segments of host photoreceptors cells. The ends of host and graft cells on the border are indicated by white and green arrows, respectively. **(H)** Continuous integration of Ku80 positive graft human RPE cells with host RPE cells, both of which expressing tight junction marker ZO-1 (arrow heads). The bottom panel shows the x–z view of the projection between the yellow dot lines of the above x–y image. **(I)** Very few cells in the graft were positive for proliferation marker Ki67 (arrow). *ONL* outer nuclear layer, *INL* inner nuclear layer, *OS* outer segment, *CH* choroid plexus, *DIC* differential interference contrast. Scale bars 100 µm (**D,D’’**,**E,G,I**), 10 µm **(F,H)**.

We transplanted hiPSC-RPE strips formed from 2 × 10^5^ cells with either 2.5 μM or 10 μM Y-27632 in albino nude rats, to test the practicality of handling during the surgical procedure, and graft survival after transplantation. The strips were successfully and stably transplanted subretinally (n = 10 with a 10 μM Y-27632 strip and n = 11 with a 2.5 μM Y-27632 strip) onto the normal RPEs in the host eyes. The grafted hiPSC-RPE strip generally looks partially flat on the fundus image (Micron IV, Phoenix Research Laboratories, Inc.), and the sectional view of the corresponding area obtained using optical coherence tomography imaging (Envisu R2200 VHR, Bioptigen, Inc.) shows a clear RPE-like reflex at the graft surface (Figs. 5A and S5A). The strips stably survived for up to 10 months without any unexpected tumor formation or graft loss, although the presence of healthy RPEs seemed to inhibit the expansion of the transplanted strip (Fig. 5B and Table S1).

The flat part of RPE-strip on color fungus image seemed corresponding to the monolayered RPE cells expanding from the graft strip in histological images (representative images from 2 different eyes presented in Fig. 5C–I and Figures S5A–E). These monolayered cells clearly expressed apical (human Ezrin) and basal (human Collagen type IV) markers, restoring the correct polarity after transplantation (Fig. 5D’,D’’). The original graft RPE-strip was often found multilayered, and the top layer randomly formed a sheet like layer also with the correct polarity (Fig. 5D,D’). These correctly polarized cells, either in a monolayer or in the top layer, also expressed phagocytosis related tyrosin-protein kinase Mer (MERTK) on the apical side and contacted host outer segments that are positive for peripherin (Fig. 5E,E’’) or rhodopsin (Fig. 5F). This suggested the presence of functional interaction between the host photoreceptors and graft RPEs. Although the apical-basal orientation seems correct when they are in a single layer or randomly when in the top layer of the graft, the graft RPEs can also form spheroids where RPE cells express apical marker Ezrin inside, or the polarity is not definite in some multilayered grafts (Figure S5F).

RPE cells in the top layer of the graft sometimes look continuous with host RPEs and contact outer segments of host photoreceptors (Fig. 5G). Monolayered graft RPE cells were also found continuous with host RPE cells and express the tight junction marker ZO-1 on the apical side (Fig. 5H). In order to assess the graft integration more closely, we directly observed the host and graft RPEs by fixing the eye 1 week after transplantation and by removal of host retina followed by ZO-1 staining (Figure S5G). Three-dimensional reconstruction of confocal microscope images implied that, in some grafted area, GFP positive graft RPEs (201B7modFucci line) can partially replace graft RPE cells, and some borderline cells were found migrating beneath the edge of the host cells (Figure S5Ga–c).

In the graft, very few cells were positive for proliferation marker Ki67 (Fig. 5I).

### hiPSC-RPE strip injection in rabbit eyes

**Figure 6:**
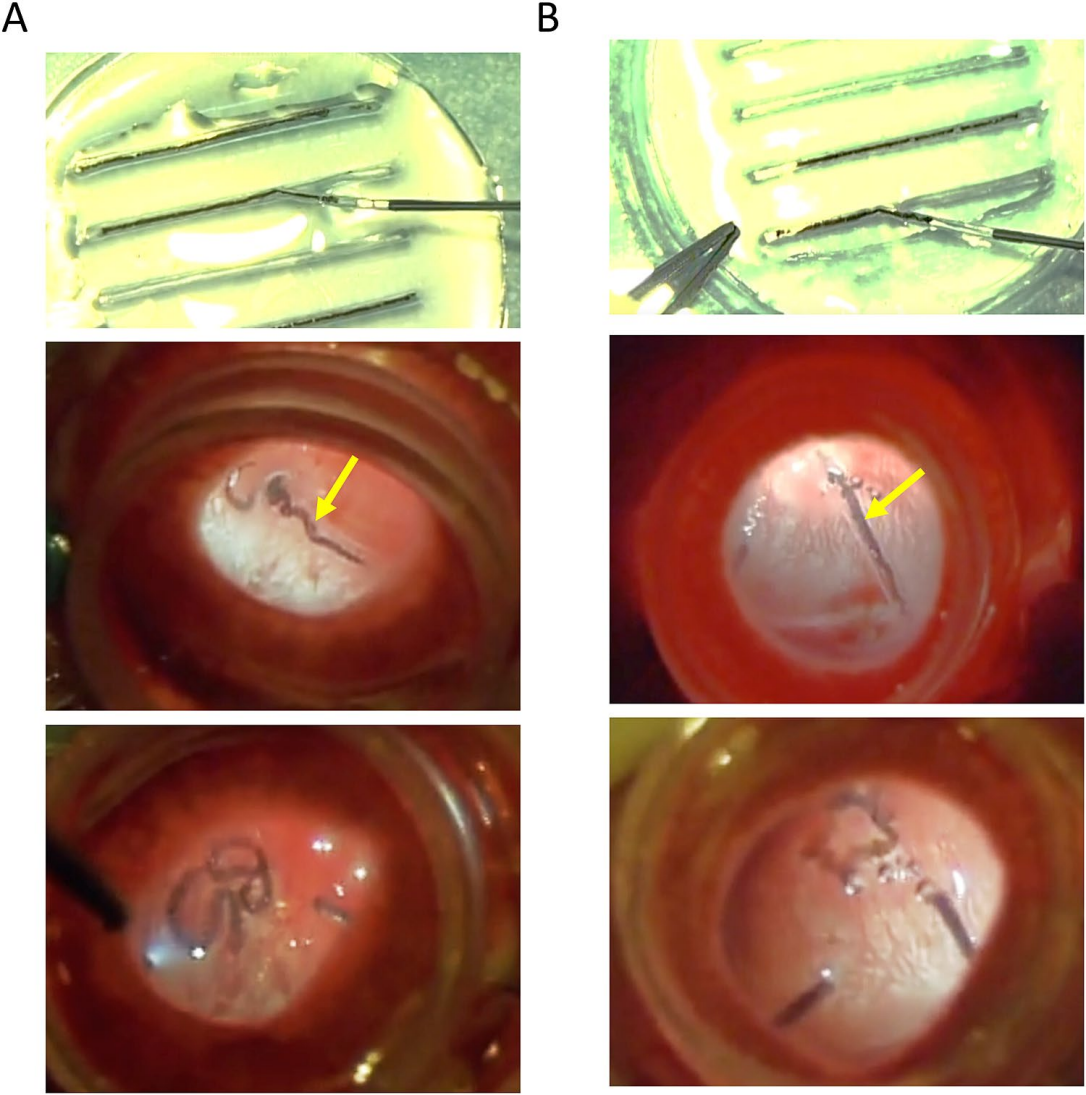
Procedure for transplantation of a hiPSC-RPE strip in two rabbit eyes. Practical handling of the hiPSC-RPE strip during the transplantation surgery was tested in two rabbit eyes **(A,B)**. Top: Loading of the hiPSC-RPE- strip. Middle: An hiPSC-RPE-strip was slowly injected into the retinal bleb. The retinal incision site is marked by a yellow arrow. Bottom: Injected hiPSC-RPE-strip. Some the surgical steps are provided as a Supplemental Movie for B.

The practicality of the surgical procedure of hiPSC-RPE strip transplantation in a clinical setting was confirmed using two rabbit eyes. An RPE strip was loaded in a 24G intravenous cannula. After routine vitrectomy, a focal retinal detachment was made, and the strip was successfully injected into the retinal bleb under good visual control. (Fig. 6 and Videos S1).

## Discussion

In this study, we succeeded in consistently inducing the formation of RPE strips in as short a period as two days, using different hiPSC-RPE cell lines. These strips were easy to handle and could be loaded into a regular 24G intravenous cannula. Once these RPE strips were placed on a non-coated culture plate, they expanded into a monolayer sheet, which exhibited the same characteristics as the original RPE cells. This observation indicates that these RPE strips could substitute for RPE sheet transplantation. For this purpose, efficient attachment of, and cell expansion from, the strip after transplantation is essential. We found that the concentration of Y-27632 affected the firmness or looseness of the strips, with a higher concentration leading to shrinkage of the strips, resulting in delayed attachment on the plate, as well as delayed expansion of the RPE cells.

The contractility of cells determines microtissue morphology under both constant curvature and adhesion strength. Higher contractility leads to condensation, and lower values lead to bridging and adhering of cells ^10,14^. In the present study, the PDMS surface seemed to provide low cell adhesiveness to enable cells to detach and condense. The PDMS-based culture device, which had a 0.2-mm curvature radius, was able to provide appropriate physical conditions for the formation and harvesting of RPE strips in a straightforward manner. The addition of Y-27632 generally suppresses contractility ^15^ and elongates the shape of human RPE ^16^. In this study, the microgroove appeared to define the direction of cell elongation, resulting in shrinkage of the strip at a higher concentration of Y-27632, as a synergistic effect between physical and pharmacological conditions. The results of experiments using different Y-27632 concentrations suggested that the adjustment of Rho kinase level was an important factor with which to control tissue formation on the concave culture surface, to improve the shape of the strip. It is also noteworthy that each hiPSC-RPE cell line may have a different optimal concentration of Y-27632.

In this study, the expansion of the RPEs from a strip appears to involve both the proliferation and migration of RPE cells, given the increased total number of cells after two weeks of expansion, and as shown by Ki67 positivity around the strip on day 7. In our scratch model experiment, the RPE cells expanded from the strips until they came in contact with resident RPE cells, at which point they appeared to stop proliferation, as again shown by Ki67 positivity, and, interestingly, formed tight junctions with the resident cells as indicated by the ZO-1 staining. This observation suggests that the RPE cells from the strip would fill the RPE defect but are not likely to overgrow. In rat experiments, we did not observe any undesired or unexpected proliferation of the graft strips, and we only observed a limited sheet-like expansion. Poor RPE expansion in vivo was considered to be due to the presence of healthy host RPEs, without any defects. Still, the monolayered graft RPEs and sometimes those in a sheet-like layer on the top of graft tissues showed the correct apical/basal polarity with the expression of phagocytosis-related MERTK on the apical side that contacts host photoreceptor outer segments, suggesting a functional interaction with host photoreceptors. The strip does not have a polarity at the time of transplantation, and it is an advantage that we can simply inject it in a surgical procedure. But this also means that currently we do not have a control over how the RPE graft cells integrate or expand. Graft RPE can also take a wrong orientation when they are multi-layered or formed spheroids. We may have to remove or disrupt the host RPEs in order to facilitate the graft expansion ideally in a monolayer, which routinely presents the correct polarity. We need an appropriate animal model to further investigate how the strip derived RPEs can efficiently expand and replace diseased RPEs in vivo.

In recent clinical studies, hESC/iPSC-RPE transplantation was conducted using either cell suspensions or sheets ^1–8^. The advantages and disadvantages of the RPE-strip approach as compared with the use of cell suspensions or sheet transplantation are summarized in Table 1. With the RPE strip, we can ensure good graft visibility during surgical procedures such as sheet transplantation, enabling good control of the graft delivery to the targeted site by preventing backflow; simultaneously, we can avoid the use of an invasive procedure, as required to make a large incision for transplantation. The RPE strips may require a supportive matrix scaffold upon which the cells can efficiently expand with the correct polarity. Cell suspension transplantation requires cells to attach and migrate, while a sheet with a scaffold can be placed on any diseased, or even remaining healthy, RPEs. The pattern of desired grafting may also favor different forms of RPEs. For instance, an RPE sheet is most suitable to cover the specific central macular area, but if RPE supplementation is required surrounding the remaining macular area, or on multiple patchy spots, multiple strips would most effectively cover these areas. Also, sheet preparation takes longer than three weeks^2,3^. The current 2-day RPE strip protocol can thus lead to a reduction in the cost incurred by using a clinical grade cell processing facility.

**Table 1:** Comparison of RPE transplantation strategies: cell suspension vs. strip vs. sheet.

	Preparation	Host condition	Surgery invasion	Visibility during surgery	Backflow leakage	Graft integration
RPE suspension	Immediate from the stock	Requires vivo scaffold	Minimal (25/38G)	Difficult	Not rare	Random
RPE strip	Minimal of 2 days	Requires vivo scaffold	Small (24G)	Good	Minimal	Random
RPE sheet	3 weeks <	No requirement for vivo scaffold	Large (> 20G)	Good	Minimal	Controlled

In conclusion, we could prepare strip-form hiPSC-RPEs, which could potentially be an optional therapeutic approach cost-wise and safety-wise, for treating some diseases involving RPE atrophy.

## Materials and methods

All animal experimental protocols were approved by the Animal Care Committee of the RIKEN Center for Biosystems Dynamics Research (BDR) and were conducted in accordance with local guidelines and the ARVO statement on the use of animals in ophthalmic and vision research. Generation of hiPS cells was approved by the ethical committee of the RIKEN Center for Biosystems Dynamics Research (BDR), and informed consent was obtained from the volunteers.

### Fabrication of PDMS-based culture device for hiPSC-RPE strip formation

The PDMS-based culture device was made with grooves of 19.5 mm length, 1 mm width, and 1.6 mm depth. The fabrication process of the device is summarized in Fig. 1A. The mold for this device was first fabricated by 3D printing using polylactic acid (PLA) filament. A 3D printed mold was shaved and coated with PDMS to adjust the shape of the grooves, and then a mold-release agent (Novec1720, Sumitomo 3M) was applied to the mold. PDMS and a curing agent for PDMS (Sylpot 184W/C) (Dow Corning Toray) were mixed in a 10: 1 ratio and poured onto the 3D printed mold and degassed for approximately 1 h. The PDMS mixture with the 3D printed mold was then placed in an 80 °C oven for 3 h. The cured PDMS was harvested from the 3D printed mold and trimmed. The curvature radius of the groove bottom was measured using a confocal scanning laser microscope (KEYENCE, VK-8710) as R 0.2 mm (Fig S1).

### Preparation of human iPSC (hiPSC) and hiPSC derived RPE cells (hiPSC-RPE)

The M8 human iPSC line was established in our laboratory by introducing six reprogramming factors (OCT3/4, SOX2, KLF4, L-MYC, LIN28, and p53 carboxy-terminal dominant-negative fragment) into peripheral blood mononuclear cells (PBMCs) from a healthy volunteer. We electroporated episomal vectors (pCE-hOCT3/4, pCE -hSK, pCE -hUL, pCE -mp53DD, pCXB-EBNA1 purchased from Addgene) to the PBMCs, and then picked iPSC-like colonies as previously described^17^. After several passages, we performed PCR analysis using the DNA extracted from the iPSC-like cells to examine whether the episomal vectors were undetectable with plasmid-specific primers (Figure S4).

201B7 and 253G1 hiPSC lines were obtained from Center for iPS Cell Research and Application, Kyoto University^18,19^. Two Fucci (Fluorescent Ubiquitination-based Cell Cycle Indicator) cell lines were made in our laboratory to visualize G1 and S/G2/M phases (M8Fucci2), and G1 and the whole cell body (201B7modFucci). Briefly, mCherry-hCdt1(30/120)-P2A-mVenus-hGem (1/110) region and mCherry-hCdt1(30/120)-P2A-mVenus region were excised from tFucci(CA)2/pCSII-EF vector (a gift from Dr. A. Miyawaki, https://cfm.brc.riken.jp/lentiviral-vectors/plasmid-list/) and ligated into the pAAVS1-Nst-CAG-DEST vector (Addgene Plasmid #80489) to make Fucci2 and modFucci donor vectors, respectively. M8 and 201B7 line was each transfected with Fucci2 or modFucci donor, respectively, with the Cas9 vector (Addgene Plasmid #62988) encoding gRNA designed to target AAVS1 locus^20^ using DNA-in CRISPR delivery media (MTI-GlobalStem, Gaithersburg, MD). After transfection, mVenus-positive M8 and 201B7 colonies were dissociated and passaged to pick single clone-oriented colonies (Figure S5).

hiPSC-RPE cells were differentiated using the SFEBq method^21–23^. (See Supplementary methods for details). Between days 30 and 60, pigmented colonies were picked and plated in 12-well plates (Corning Incorporated, Corning, NY) coated with iMatrix 511 (Nippi. Inc., Tokyo, Japan) containing a 1: 1 “Mixed medium” of "RPE adhesion medium" and "RPE maintenance medium". “RPE adhesion medium” contains DMEM/F-12 (Sigma-Aldrich, St. Louis, MO), 10% fetal bovine serum (SAFC Biosciences Inc.), gentamicin solution (Sigma-Aldrich) and “RPE maintenance medium” contains DMEM-low glucose (Sigma-Aldrich), 30% F-12(Sigma-Aldrich), 2% l-glutamine solution (Sigma-Aldrich), 2% B-27™ Supplement (50×) (Thermo Fisher Scientific Inc., Waltham, MA), and gentamicin solution (Sigma-Aldrich). Once the cells were attached, the medium was changed to RPE maintenance medium with 10 ng/mL basic fibroblast growth factor (bFGF) (Wako, Osaka, Japan). The cell culture was expanded in the RPE maintenance medium through one passage, and the cells were stocked at − 150 °C using Stem-Cell Banker (Nippon Zenyaku Kogyo Co., Ltd.).

For each experiment, the stocked cells were thawed and plated with the mixed medium, and then the medium was changed to RPE maintenance medium supplemented with 10 ng/mL bFGF and 0.5 µM SB431542 (Sigma-Aldrich), which was changed every few days until the cells were used. RPE cells differentiated from 253G1 cells were prepared as described previously ^24^.

### Formation of hiPSC-RPE strips

After thawing, hiPSC-RPE cells were cultured for two weeks, and then collected and plated in each groove of the PDMS-based culture device at the cell number described in each experiment, with15 µl RPE maintenance medium containing Y-27632(Wako) (concentration indicated in each experiment).

### Analysis of expansion area of RPEs from a plated hiPSC-RPE strip

hiPSC-RPE strips were released from a groove in the device and placed on non-coated 24-well plates (Corning) with a strip/well in mixed medium, that was changed to RPE maintenance medium with bFGF and SB431542 after strip attachment two to three days later. The dishes in culture were photographed using an IncuCyte Zoom system (Essen BioScience). From the image data, expanded cells were manually marked with Fiji image analysis software, and the expanded area was measured.

### In vitro RPE damaged model

Confluent RPE cultures in 24-well plates were treated with MitomycinC (Kyowa Hakko Kirin, Tokyo, Japan) at 6 µg/mL. On the following day, a band of RPE cells was scratched and removed using a cell scraper of approximately 6 mm width. hiPSC-RPEs of either cell suspension (2 × 10^5) or of a strip form was plated (“transplanted”) over or in the scratched RPE area. The cells were maintained in 0.5 ml medium and were collected for ELISA at the time of each intervention, with 24 h incubation before medium change. The schedule of the experiment plans and the timing of medium collection are shown in Fig. 4A.

### ELIZA for VEGF and PEDF

VEGF and PEDF were performed according to the manufacturer’s protocol of the VEGF Human ELISA Kits (Invitrogen, Thermo Fisher Scientific Inc., Waltham, MA) and Human PEDF ELISA Kits (BioVendor, Brno, Czech Republic), respectively.

### Transplantation in rat eyes

Immune-deficient F344/NJc1-rnu/rnu female nude rats, six weeks old, were obtained from CLEA Japan. Disposable micropipettes (1-000-0500, Drummon, Alabama, USA) were pulled using a micropipette puller (P-97/IVF Puller, Sutter Instrument, California, USA), and the tip was cut and sharpened using a microgrinder (EG-400, Narishige, Tokyo, Japan). The micropipettes were then adapted to a microelectrode holder (MPH310, World Precision Instruments., FL, USA) on a 6.3 mm electrode handle (2505, World Precision Instruments.), connected to a 10 µl micro-syringe (1701LT, Hamilton, MA, USA) with an extension tube. The animals were anesthetized by inhalation of 5% isoflurane in air, and the eyes were dilated with 0.4% tropicamide. The hiPSC-RPE strip was loaded in a micropipette and the strip was transplanted subretinally.

### Transplantation in rabbit eyes

kbl:JW rabbits, nine weeks old, were obtained from Oriental Yeast Co., Tokyo. The animals were anesthetized by intramuscular injection of 60 mg/kg ketamine and 10 mg/kg xylazine. After a routine vitrectomy with posterior vitreous detachment, the iPSC-RPE strip was loaded into the outer tube of a 24-gauge indwelling needle cannula (SS-6, TOP Corporation, Tokyo, Japan) supported by a custom-made flat-cut 25G needle inside. A focal retinal detachment was made with a PolyTip Cannula 25 g/38 g (MedOne, FL, USA), and the graft strip was slowly injected into the detached retinal bleb using a 50 µl micro-syringe (1705LT, Hamilton). Then, perfluorocarbon liquid (8065900111, Alcon, Inc., Geneva, Switzerland) was injected over the detachment to press and attach to the retina, followed by fluid-gas exchange.

## Supplementary Information

Supplementary Information.

Supplementary Video S1.
