# Supplementary material for: Human iPS cell derived RPE strips for secure delivery of graft cells at a target place with minimal surgical invasion
Source: Sci Rep. 2021 Nov 2;11:21421. doi: 10.1038/s41598-021-00703-x (PMC8563929; doi:10.1038/s41598-021-00703-x)
Supplement: Supplementary file 1 — Supplementary Information. [file 41598_2021_703_MOESM1_ESM.pdf]

SUPPLEMENTARY INFORMATION

Human iPS cell derived RPE strips for secure delivery of graft cells at a target place with minimal surgical invasion.

Mitsuhiro Nishida[1], Yuji Tanaka[1, 2], Yo Tanaka[3], Satoshi Amaya[3], Nobuyuki Tanaka[3], Hirofumi Uyama[1,4], Tomohiro Masuda[1,6], Akishi Onishi[1], Junki Sho[1,5], Satoshi Yokota[1,4], Masayo Takahashi[1,4,5,6], Michiko Mandai[1,4,]

supplemental methods

- Differentiation of iPSC-RPEs
- Immunohistochemistry
- Polymerase chain reaction (PCR) for RPE marker genes

supplemental Figure

- Figure S1: Measurement results of PDMS grove structures.
- Figure S2: Optimization of hiPSC-RPE strip formation.
- Figure S3: Optimization of Y-27632 concentration for hiPSC-RPE strip formation.
- Figure S4: Proliferation of RPE strips after plating.
- Figure S5: Graft RPE integration after transplantation.
- Figure S6: Features of M8 hiPSC line.
- Figure S7: Construction of M8Fucci2 and 201B7modFucci line.
- Figure S8: Photo-images of whole gels in Figure 3D.

supplemental Table1

- Table S1: Number of rats for each transplantation conditions and observation period.
- Table S2: Used antibody information.
- Table S3: Primers for RPE markers

Videos-S1

- hiPSC-RPE strip injection in a rabbit eye

Supplementary methods

Differentiation of iPSC-RPEs

hiPSC-RPE cells were differentiated using the SFEBq method as described by Kuwahara et al.[1-3] In brief, 5μM SB431542 (S4317, Sigma-Aldrich, St. Louis, MO) and 300nM SAG (ALX-270-426-M001, Enzo Life Sciences, Inc, NY, US) were added to the iPS cells the day before the induction of differentiation. On day 0, the iPS cells were suspended in growth factor-free chemically defined medium (gfCDM) containing IMDM: F12 (1:1), 10% Knockout Serum Replacement (10828-010, Thermo Fisher Scientific Inc., Waltham, MA), 1% Chemically Defined Lipid Concentrate (11905031, Gibco, Thermo Fisher Scientific Inc., Waltham, MA), 30w/v % BSA (017-22231, FUJIFILM Wako Pure Chemical Corporation, Osaka, Japan), 450μM monothioglycerol (M6145, Sigma-Aldrich, St. Louis, MO), and penicillin-streptomycin, supplemented with 30nM SAG, 20μM Y-27632 (259-00613, FUJIFILM Wako Pure Chemical Corporation, Osaka, Japan) and were cultured in Prime surface 96V (MS-9096V, Sumitomo Bakelite Co., Ltd., Tokyo, Japan). 1.5nM BMP-4(314-BP, R&D System, Inc. minneapolis, mn) was added on day 6, and half the gfCDM was changed every 3 days. On day 18, the cells were transferred to Ultra Low Culture Dish (3262, Corning Incorporated, Corning, NY) with DMEM/F12-Glutamax medium (10565-018, Gibco, Thermo Fisher Scientific Inc., Waltham, MA) containing 1% N2 supplement (17502048, Gibco, Thermo Fisher Scientific Inc., Waltham, MA), 3 μM CultureSure CHIR99021 (034-23103, FUJIFILM Wako Pure Chemical Corporation, Osaka, Japan), and 5 μM SU5402 (SML0443-5MG, Sigma-Aldrich, St. Louis, MO). On day 22 and after, the cells were cultured in DMEM/F12-Glutamax medium with 1% N2 supplement, 10% Fetal Bovine Serum (FB-1365/500, Biosera Inc, Manila, Philippines), 0.5 μM retinoic acid retinoic acid (R2625, Merck, Sigma-Aldrich, St. Louis, MO), 0.1 mM taurine (T-0625-10G, Sigma-Aldrich, St. Louis, MO), and 1x antibiotic antimycotic (15240062, Thermo Fisher Scientific Inc., Waltham, MA).

Immunohistochemistry

Strips or cells in culture were fixed with 4% paraformaldehyde at room temperature for 15 min. Transplanted eyes were fixed with 4% paraformaldehyde at 4 °C for 1 h, and the cornea was removed after fixation. Samples for frozen sections were soaked in 30% sucrose for one day or more, and frozen in OCT compound (Sakura Finetek Japan Co., Ltd.) for cryo-sectioning of 10 μm thickness. Samples were permeabilized with 0.2% Triton X -100 in phosphate-buffered saline for 30 min, blocked with Blocking One (Nacalai Tesque, Kyoto, Japan) for 1 h at room temperature, and incubated with primary antibodies diluted in Antibody Diluent (Dako, Glostrup, Denmark) overnight at 4 °C. The antibodies used are listed in Table S2. Treatment with secondary antibodies was conducted for 1 h at room temperature, and images were obtained using a confocal microscope (LSM700; Carl Zeiss, Leica-TCS SP8). and fluorescence microscope (BZ-X810; KEYENCE).

Polymerase chain reaction (PCR) for RPE marker genes

Total RNA was extracted from the cells using RNeasy Micro Kits (Qiagen, Valencia, CA, and cDNA was synthesized using SuperScript® III Reverse Transcriptase Kits (Invitrogen). The sequences of the primers for the RPE markers (BEST1, RPE65, and CRALBP) and the housekeeping gene (GAPDH) are listed in Table S3. PCR reactions were performed using Blend Taq® -Plus- (TOYOBO Co.,Ltd, Osaka, Japan). Thermal cycling conditions were as follows: one cycle at 94 °C for 180 s; 32 cycles of denaturing at 94 °C for 30 s, priming at 58 °C for 30 s, and extension at 72 °C for 60 s, followed by one cycle at 72 °C for 60 s.

References

１. Nakano, T. et al. Self-formation of optic cups and storable stratified neural retina from human ESCs. Cell Stem Cell 10, 771–785 (2012).

２. Kuwahara, A. et al. Generation of a ciliary margin-like stem cell niche from self-organizing human retinal tissue. Nat. Commun. 6, (2015).

３. Kuwahara, A. et al. Preconditioning the Initial State of Feeder-free Human Pluripotent Stem Cells Promotes Self-formation of Three-dimensional Retinal Tissue. Sci. Rep. 9, 1–16 (2019)

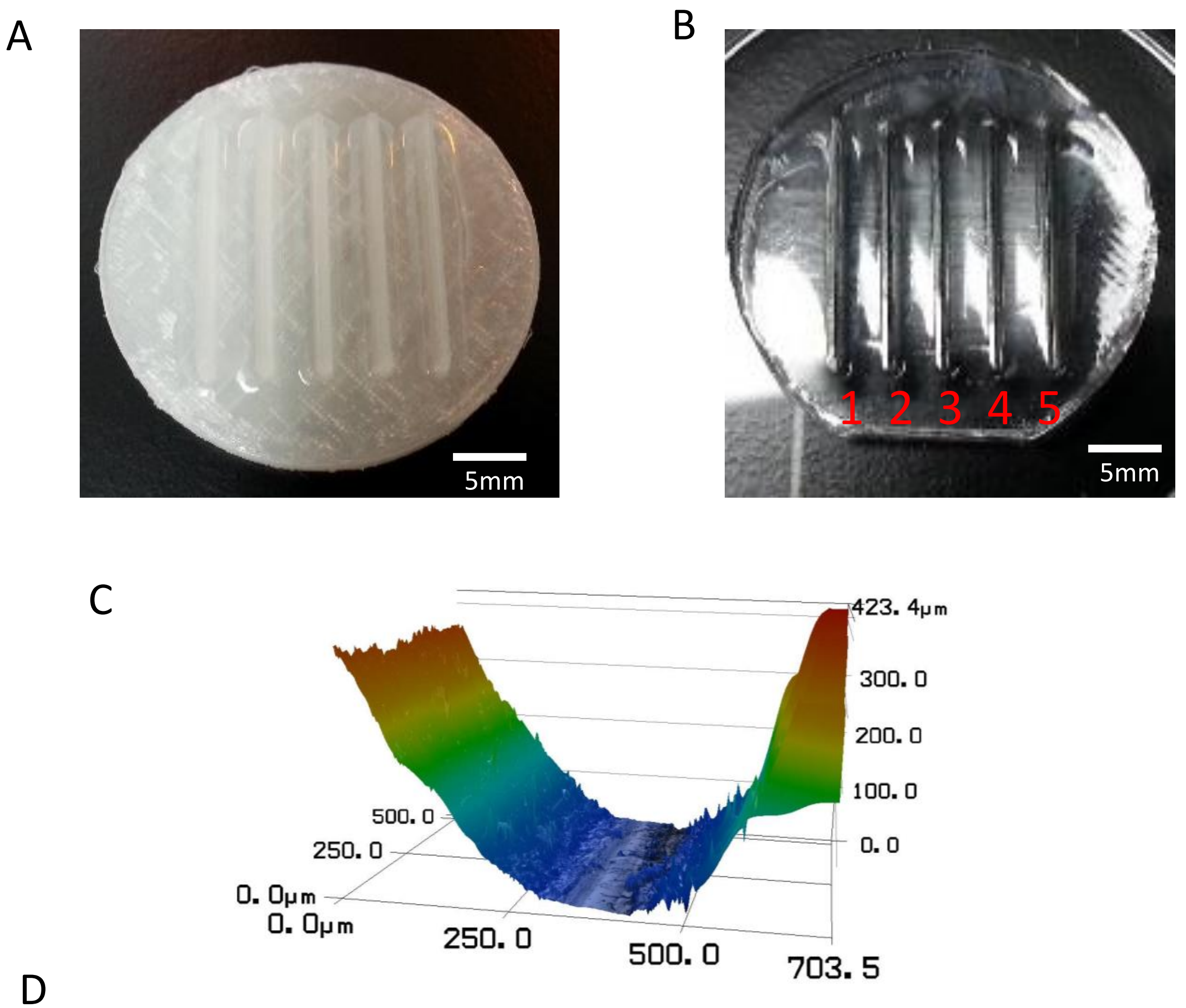

D

	R (bottom) [mm]	Width [mm]	Depth [mm]	Length [mm]
1	0.32	1.13	1.59	19.5
2	0.13	1.09	1.58	19.5
3	0.09	1.23	1.59	19.5
4	0.20	1.13	1.61	19.5
5	0.29	1.21	1.66	19.5
Ave.	0.21	1.16	1.60	19.5

Figure S1: Measurement results of PDMS grove structures.
A: A picture of a PDMS coated mold, which has 5 fins with 19.5 mm long and 1.6 mm height
B: A picture of a PDMS based culture device for groove structure measurement, which has 5 grooves with 19.5 mm long 1 mm wide and 1.6 mm depth
C: Measured image of a groove bottom with a laser microscope
D: Measured groove shape for a PDMS based culture device. The average values for each measurement item are curvature radius of bottom surface 0.21 mm, groove width 1.16 mm and groove depth 1.60 mm

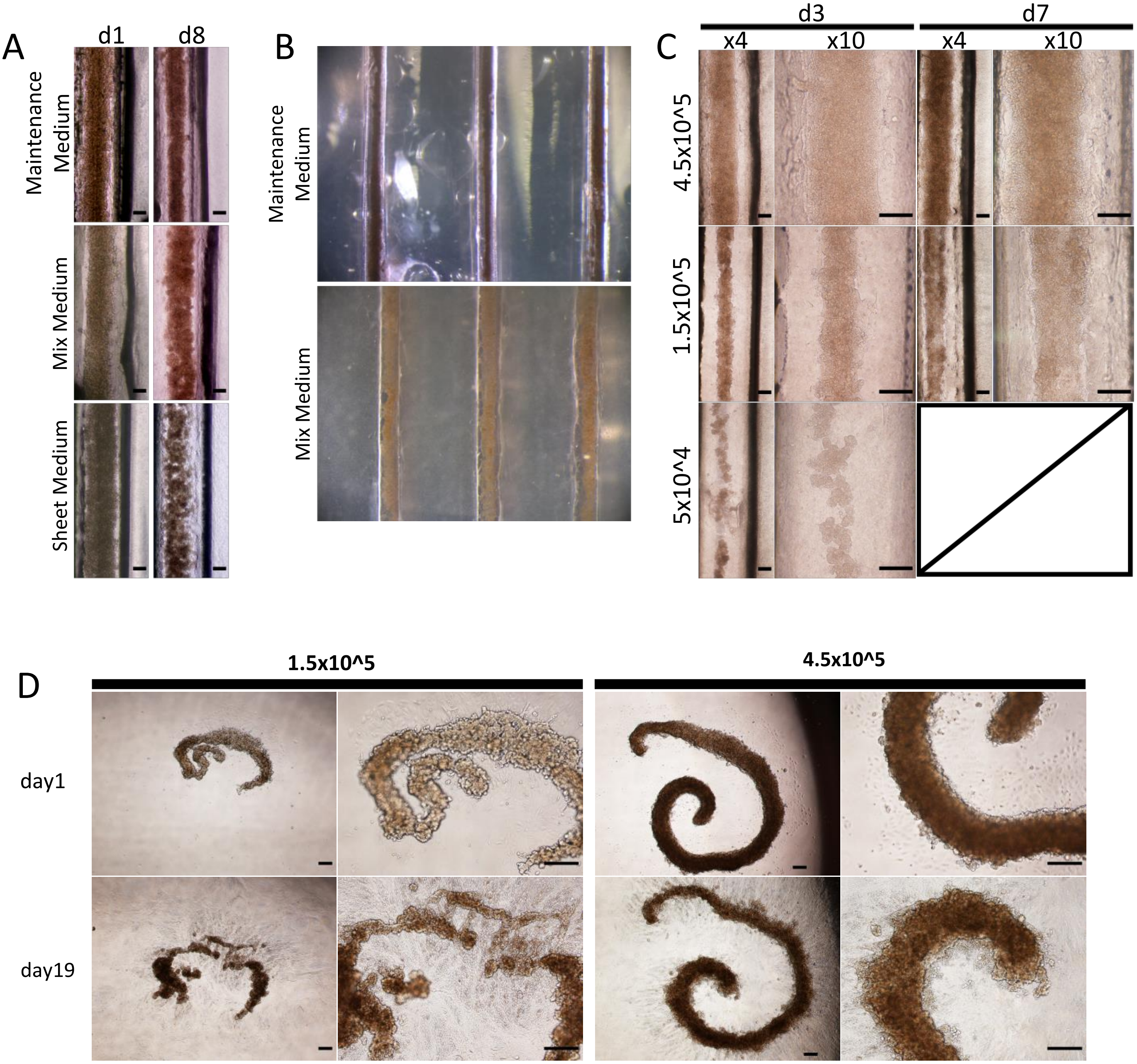

Figure S2: Optimization of hiPSC-RPE strip formation.
A: hiPSC-RPE cells were plated in the grooves of the mold with three different types of medium with 10μM Y-27632 (Sheet Medium, Mix Medium , Maintenance Medium)
B: hiPSC-RPE strip appearance in the mold (Mix Medium , Maintenance Medium)
C: hiPSC-RPE cells were plated at 4.5x10^5 , 1.5x10^5 , and 5x10^4 in a groove and were incubated for 3 and 7 days (day3 , day7)
D: The appearance of hiPSC-RPE strip formed with 1.5x10^5 or 4.5x10^5 placed on a plate for 19 days.
Scale bars 200μm(A, C, D)

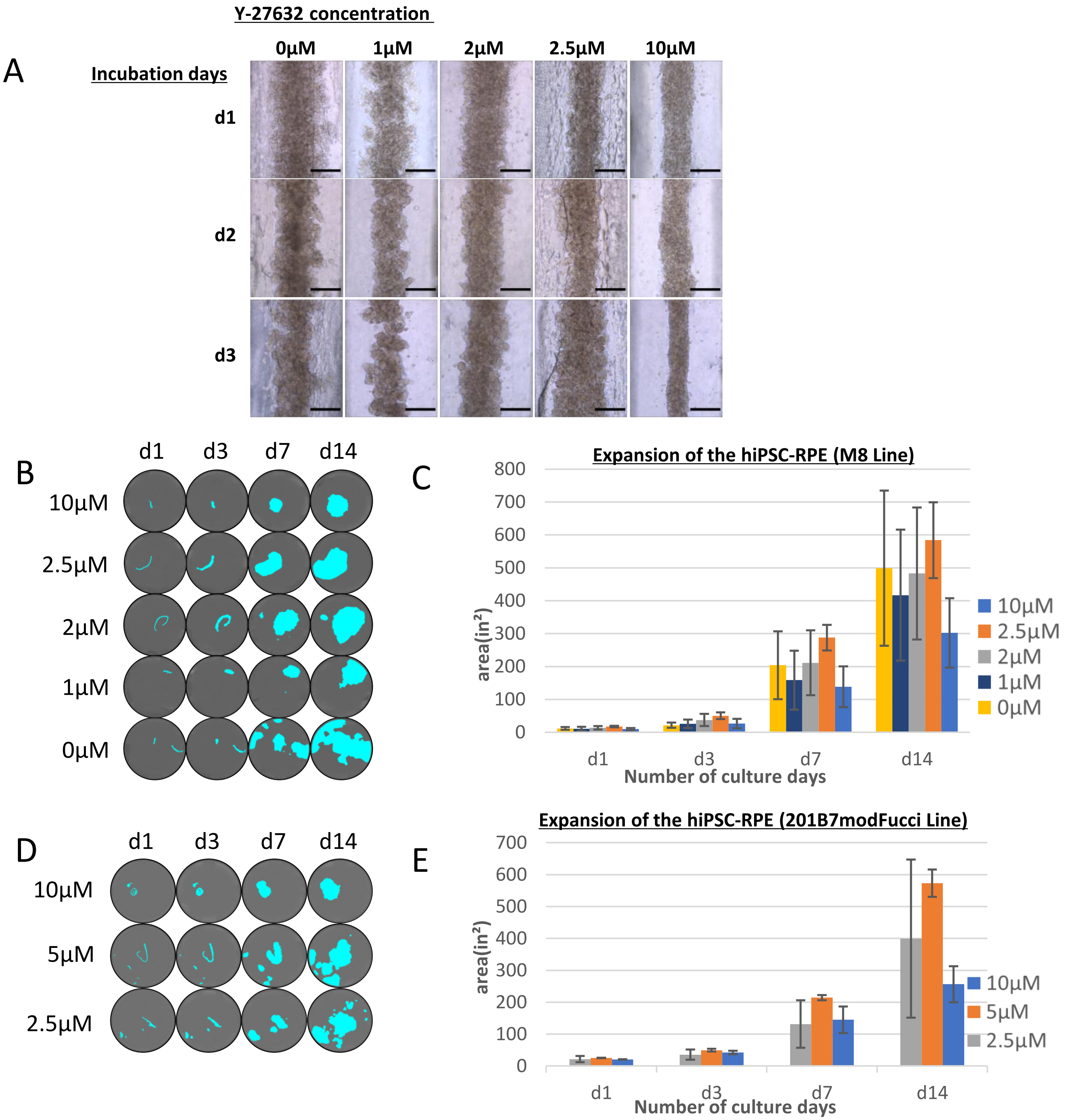

Figure S3: Optimization of Y-27632 concentration for hiPSC-RPE strip formation.

A: Strip formation under different Y-27632 concentrations

B: Strips （M8 Line） were placed in a 24 well plate and the expansion of the coverage area by hiPSC-RPE was monitored. The strip with 2-2.5 µM Y-27632 was most adhesive to the plate and tended to attach flat when placed on the well.

C: Expansion of the hiPSC-RPE （M8 Line） covered area after placing a hiPSC RPE strip in each well (n=6 for each Y-27632 concentration)

D: Strips （201B7modFucci Line） of day 2 were placed in a 24 well plate and the expansion of the coverage area by hiPSC-RPE was monitored. The strip with 2.5-5 µM Y-27632 was consistently adhesive to the plate and tended to attach flat when placed on the well.

E: Expansion of the hiPSC-RPE（201B7modFucci Line） covered area after placing a hiPSC RPE strip in each well (n=2 for each Y-27632 concentration)

Data are presented as means ± SD. Scale bars 200µm(A)

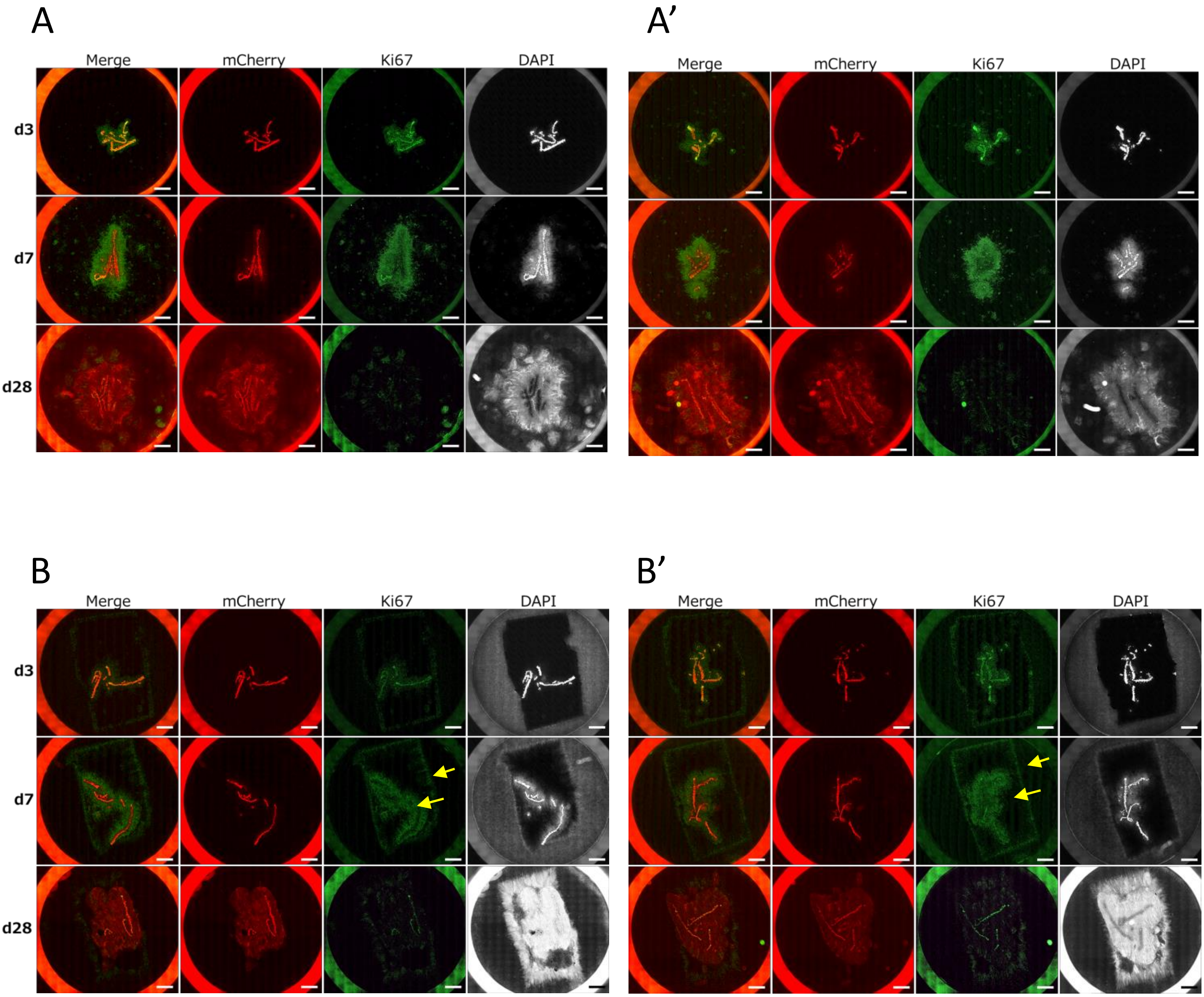

Figure S4: Proliferation of RPE strips after plating

201B7ModFucci RPE strip (mCherry positive) was plated on a non-coated dish (AA’) and with a partial RPE removal (BB’) and these were fixed on day3, 7, and 28 for immunostaining for Ki67. Ki 67 positive cells were observed on and around the margins of pre-plated RPEs and an RPE strip on day 7 (arrows), which became less evident on day 28 when the plate was almost confluent with or without RPE removal. Scale bars=5000μm

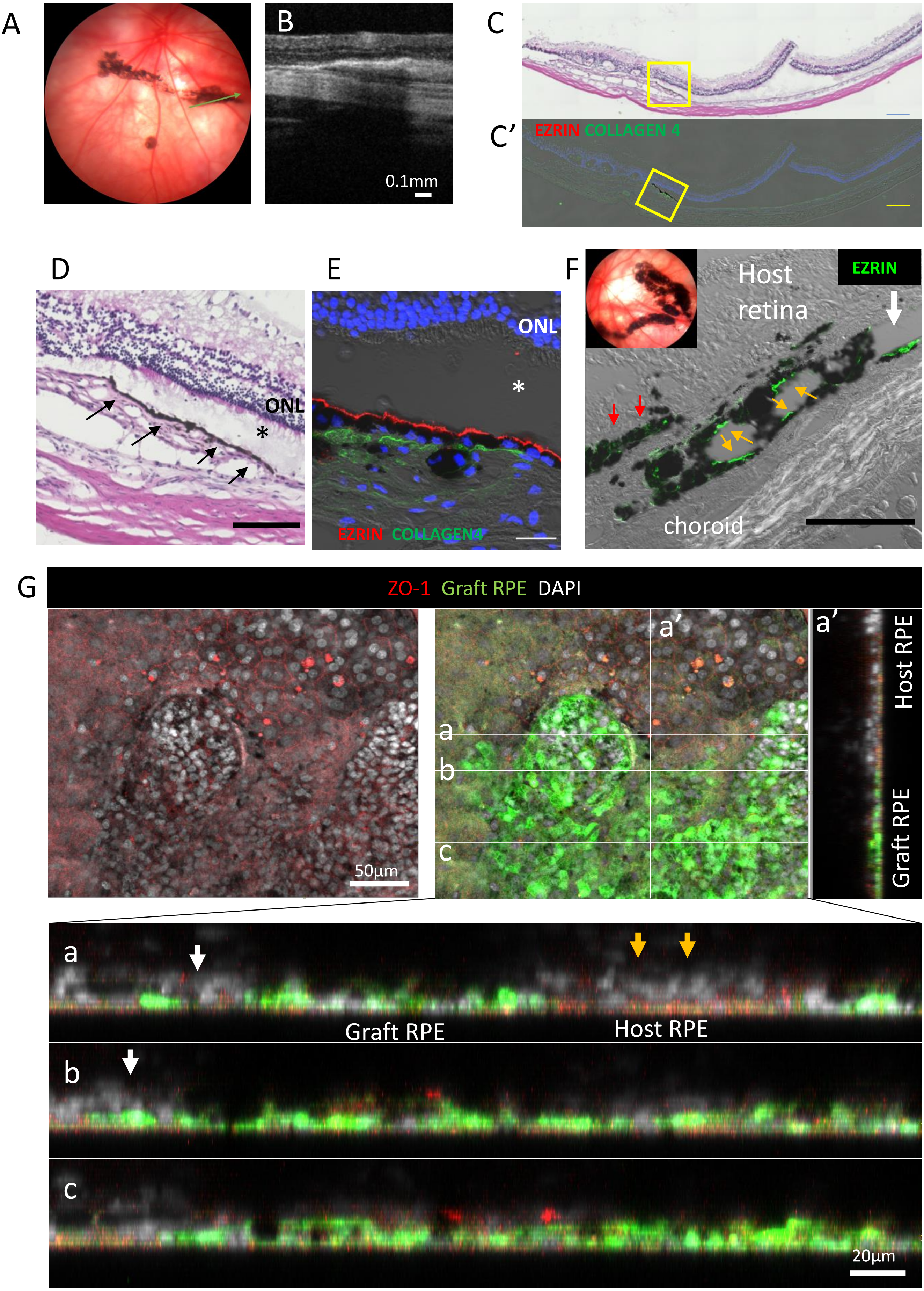

A
B
0.1mm
C
C'
EZRIN COLLAGEN 4
D
ONL
*
E
ONL
*
EZRIN COLLAGEN4
F
Host
retina
EZRIN
choroid
G
ZO-1 Graft RPE DAPI
50μm
a'
a
b
c
a'
Host RPE
Graft RPE
a
Graft RPE
Host RPE
b
c
20μm

Figure S5 Graft RPE integration after transplantation

A,B: Vivo imaging of a sample 3 months after transplantation. OCT sectional view (B) at the arrow line in A.
C-C'; Hematoxylin and eosin staining and immunolabeling of the apical and basal markers, human-specific ezrin and collagen type IV on serial sections from the eye in A..
D: A magnified view of the box in C. Monolayered pigmented RPEs （arrows） face outer segments of host photoreceptors (asterisk).
E: A magnified view of the square box in C'. Grafted RPEs showed correct polarity, as shown by the apical and basal markers human-specific Ezrin and Collagen type IV, which faces the outer segments on host photoreceptor cells (white asterisk).
F: Graft hiPSC-RPE presenting the multilayer with undefined polarity (red arrow), spheroids with apical side inward (yellow arrows) alongside the monolayered RPE with the correct polarity (white arrow) from the eye in color fundus image on top left.
G: A direct observation of host-graft RPE interaction 1 week after transplantation of the hiPSC-RPE-strip in an albino rat eye with ZO-1 staining after removal of overlying retina. ZO-1 positive host RPEs on X-Y plane confocal image is partially replaced by GFP positive graft cells (201B7ModFucci). Reconstructed Z-stack sectional views at the lines a-c and a' are shown on the bottom and side panels. GFP positive graft cells lined up with host RPE, and some graft cells on the borderline are migrating beneath the host RPE (white arrows). Host RPE cells were partially double layered near the host graft border (orange arrows)
ONL, outer nuclear layer
Scale bars 200 µm (C,C'), 100 µm (D, F) , 20 µm (E)

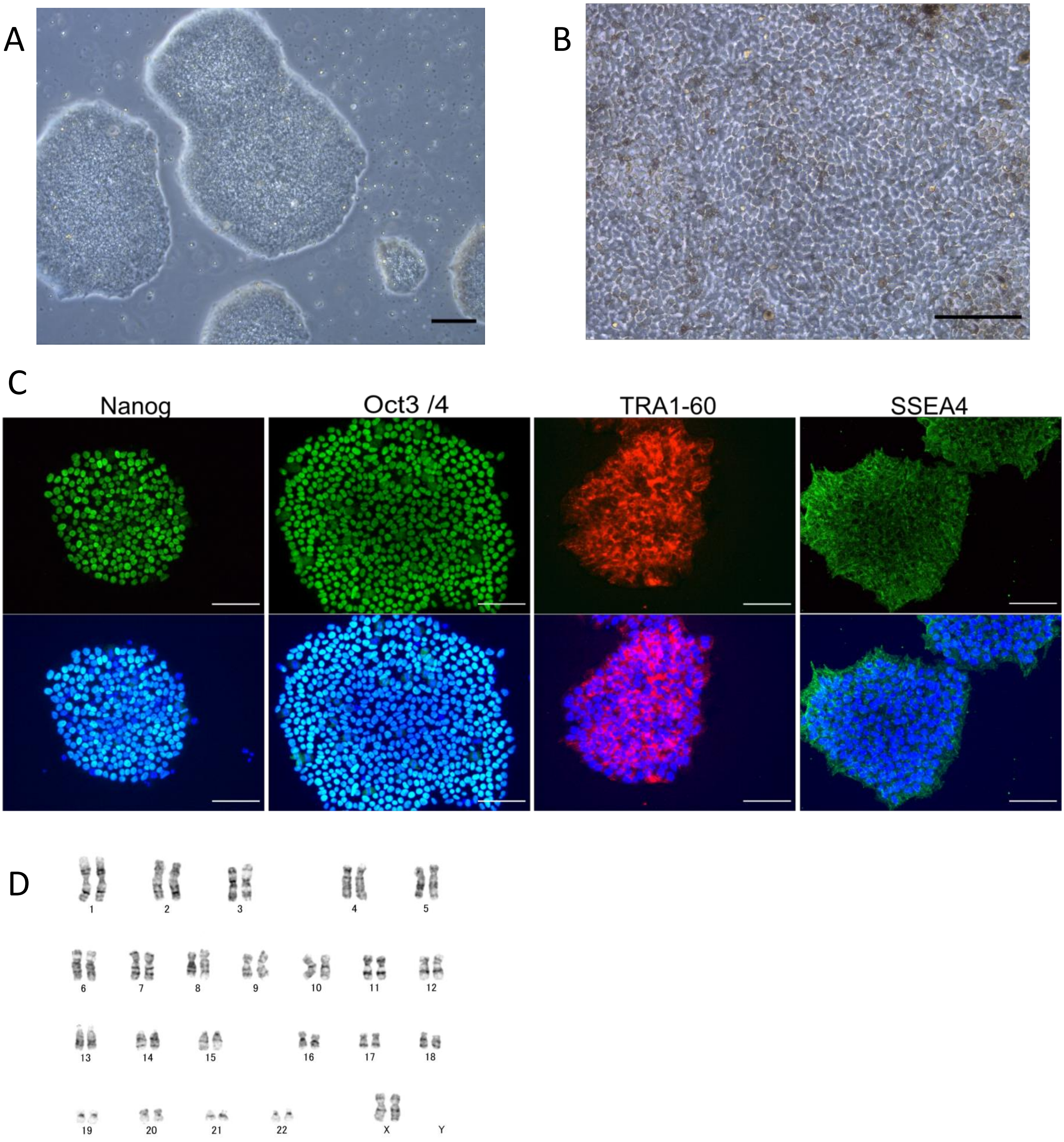

Figure S6: Features of M8 hiPSC line.

A:iPS clolonies of M8 line

B: Differentiate RPE cells from M8 hiPSC line.

C: Expression of iPSC markers in M8 iPSC colonies. Lower column shows the merged images with DAPI staining

D: Karyotype analysis of M8 hiPS cells.

Scale bars 200μm (A), 100μm(B, C)

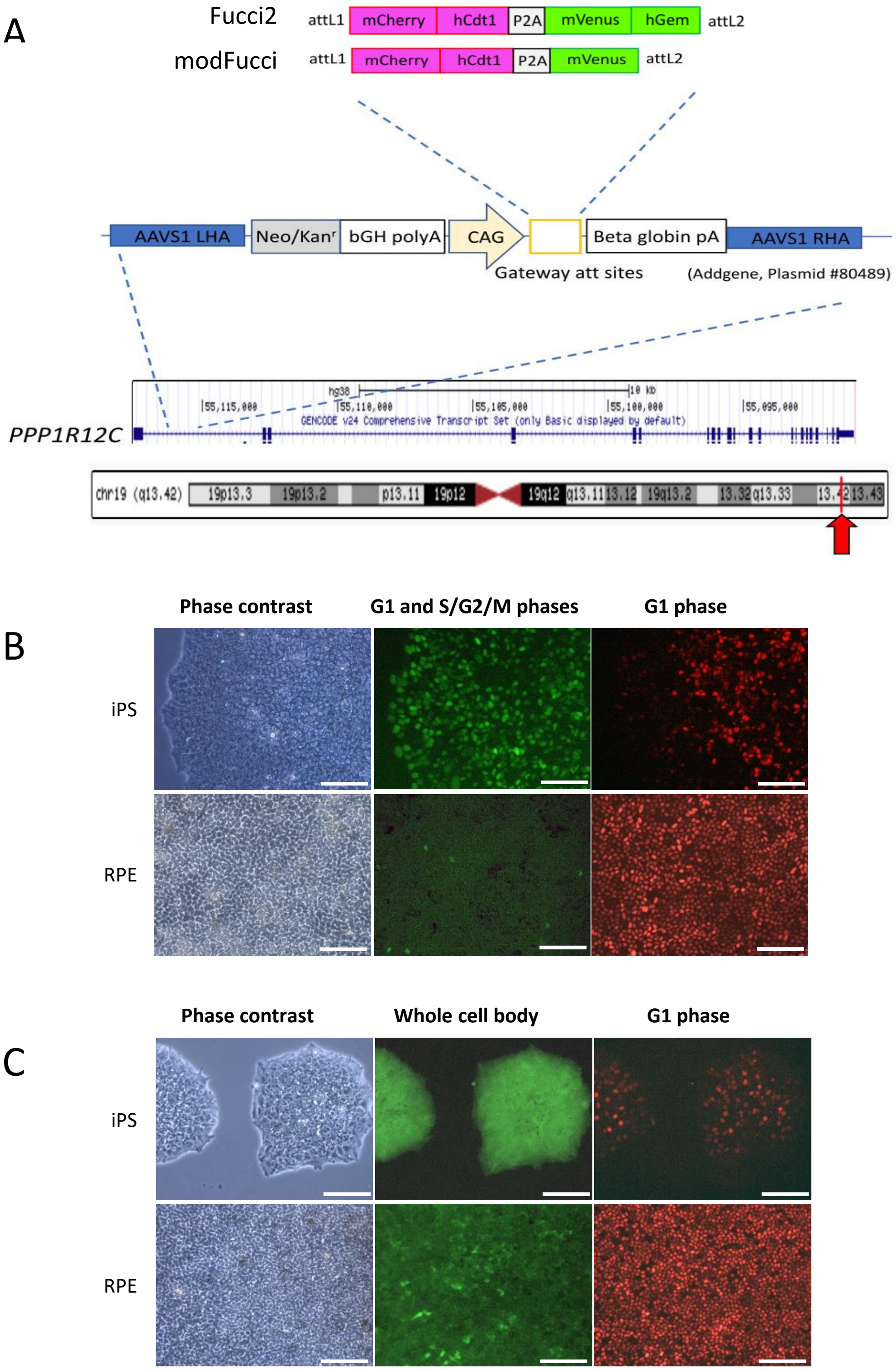

Figure S7: Construction of M8Fucci2 and 201B7modFucci line.

A: A diagram of the strategy to introduce Fucci2 and modified Fucci construct into the AAVS1 locus.

B: M8Fucci2 line can visualize the G1 and S/G2/M phases (mVenus) and the G1 phase (mCherry) of iPSCs (above) and RPE cells differentiated from the M8Fucci2 line (below).

C: 201B7modFucci line can visualize the whole cell body (mVenus) and the G1 phase (mCherry) of iPSCs (above) and RPE cells differentiated from the 201B7modFucci line (below).

Scale bars 100µm (B, C)

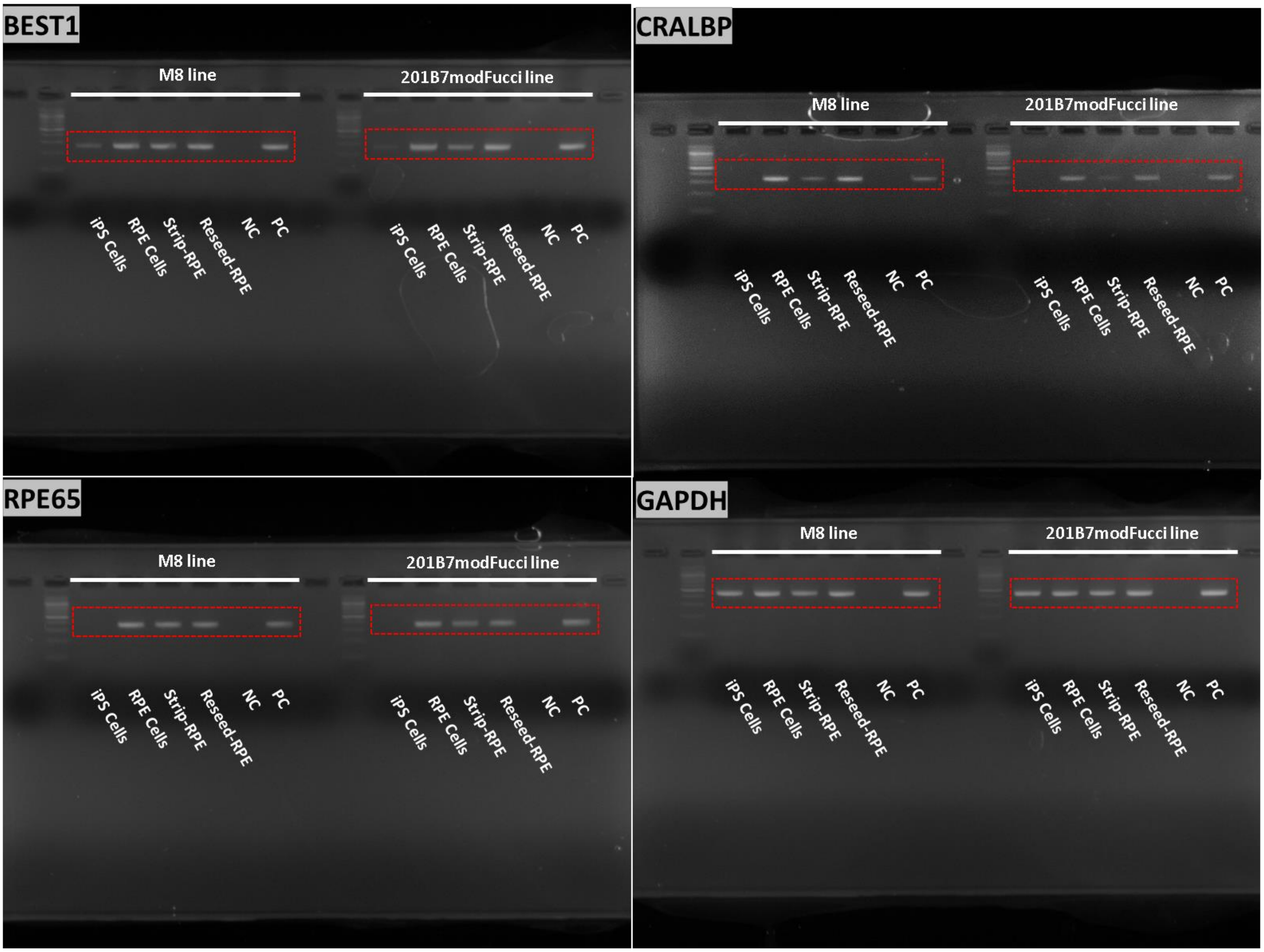

Figure S8: Photo-images of whole gels in Figure 3D

SUPPLEMENTARY TABLES

Table S1: Number of rats for each transplantation conditions and observation period.

		observation period			
concentraiton of Y-27632	cell number to form a strip	1M	2M	3M	8-10M
10μM	200000	3	2	2	3
2.5μM	200000	3	2	2	4

Table S2: Used antibody information.

Antigen	Host	Company	Product	Dilution
collagen type IV	Mouse	abcam	Ab6311	1:400
Ezrin	Rabbit	R&D Systems	MAB7239	1:1000
ZO-1	Mouse	Invitrogen	33-9100	1:1000
ZO-1	Rabbit	Invitrogen	61-7300	1:500
Laminin	Rabbit	Abcam	Ab11575	1:400
MiTF	Mouse	Abcam	Ab80651	1:500
RPE65	Mouse	Millipore	MAB5428	1:500
MERTK	Rabbit	abcam	ab52968	1:500
Ku80/XRCC5	Goat	R&D Systems	AF5619	1:200
CD147	Goat	R&D Systems	AF972-SP	1:200
Ki67	Mouse	BD Biosciences	550609	1:200
Peripherin	Rabbit	Proteintech	18109-1-AP	1:1000
Peripherin	Goat	Santacruz	sc-18946	1:500
Rhodopsin	Mouse	sigma	O4886	1:500

Table S3: Primers for RPE markers

Gene		Sequence (5→' 3')
BEST1	F	5'-TAGAACCATCAGCGCCGTC-3'
	R	5'-TGAGTGTAGTGTGTATGTTGG-3'
RPE65	F	5'-TCCCCAATACAACTGCCACT-3'
	R	5'-CCTTGGCATTCAGAATCAGG-3'
CRALBP	F	5'-GAGGGTGCAAGAGAAGGACA-3'
	R	5'-TGCAGAAGCCATTGATTTGA-3'
MERTK	F	5'-TCCTTGGCCATCAGAAAAAG-3'
	R	5'-CATTTGGGTGGCTGAAGTCT-3'
GAPDH	F	5'-ACCACAGTCCATGCCATCAC-3'
	R	5'-TCCACCACCCTGTTGCTGTA-3'
